# Editorial: Healthy foods and dietary patterns in modern consumer

**DOI:** 10.3389/fnut.2023.1239488

**Published:** 2023-08-18

**Authors:** Prisco Piscitelli, Annamaria Colao

**Affiliations:** UNESCO Chair on Health Education and Sustainable Development, Federico II University, Naples, Italy

**Keywords:** foods, health, label, Mediterranean diet, Nutriscore, Med Index, Planeterranean diet

According to the ancient Greek philosophers, “*we are what we eat*.” Nowadays, obesity and overweight, even in young people and children, represent a serious public health problem, reaching epidemic proportions all over the world (the “new pandemic of the twenty first century,” as defined by the World Health Organization). There is a clear link between nutritional habits and body weight, as well as with natural mortality, type II diabetes, cognitive function, and other conditions. These topics have been addressed in the Research Topic by Almoraie et al. (obesity), Brayner et al. (diabetes), Gou et al. (cognitive function), Goulart et al. (cognitive performance), Kim et al. (metabolic syndrome), Liu et al. (nutritional patterns and mortality), and Xi et al. (sodium intake), while Trabelsi et al. gave an original insight into temporary fasting.

On the other issue, the Research Topic of nutritional deficiencies in relation to locally available foods and their ability to meet nutrient requirements has been recently highlighted by the scientific community as a threat to low-income countries. As highlighted by Foroozanfar et al. in their article about social determinants of nutritional behaviors, it is noteworthy that food and the way we eat are a major driver of our health. This explains the increasing interest of the general public toward the health properties of food and labeling regulations that are able to foster informed nutritional choices from the perspective of modern consumer-friendly policies, as highlighted in the article by Badji et al.. The issue is so crucial that some international institutions—such as the European Union—are assessing the possibility of adopting unified labeling systems to foster appropriate food choices by consumers. For this purpose, the so called “Nutriscore” (1) has been proposed as a front-of-pack labeling system (FOPL), which was created by the French Nutritional Epidemiology Research Team (EREN) on the basis of nutritional scores elaborated by the Food Standards Agency in the UK. More recently, a new FOPL based on the “One Health” approach named “Med Index” (2) has been released, which was created by the University of Bari in Italy along with the Italian Society of Environmental Medicine SIMA and officially presented at the 1st Yale Gastronomy and Culture Symposium in Crete, 3–5 May 2023. Med Index has been designed to provide information not only about calories and fats (as the Nutriscore does) but also to summarize on a simple label the healthy nutritional properties of the food, the physical activity needed to burn the energy gained by eating it, the sustainability of production processes that are behind the food, and the social responsibility of the companies. Interestingly, Med Index does not require food producers to perform new efforts, but it is based on the certifications already acquired by the companies concerning both the dimensions of sustainable production and social responsibility. These two scores might possibly integrate different kinds of information if used in combination, thus avoiding possible confounding messages for the consumers. In particular, the 4th International Yale Symposium on Olive Oil and Health (held in Rome, September 15–18, 2022) had already endorsed a position statement on the issue of the NutriScore use and front-of-package label (FOPL) for olive oil, recommending that—based on the constantly accumulating evidence of its health benefits (3)—olive oil should either be labeled as a Green-A-grade food by NutriScore or be included in the highest category within any FOPL system (or as an alternative recommending its exclusion from the NutriScore). The reason for that is mainly related to the labeling of olive oil with the NutriScore level B/C, which would undoubtedly create confusion and dampen the trust of consumers in European guidance and regulations, as it is in clear contrast to the EFSA health claim [Commission Regulation (EU) 432/2012]. This health claim unequivocally emphasizes the health benefits of olive oil: “olive oil polyphenols contribute to the protection of blood lipids from oxidative stress. The claim may be used only for olive oil, containing at least 5 mg of hydroxytyrosol and its derivatives (e.g., oleuropein complex and tyrosol) per 20 g of olive oil. In order to bear the claim information shall be given to the consumer that the beneficial effect is obtained with a daily intake of 20 g of olive oil. Replacing saturated fats in the diet with unsaturated fats contributes to the maintenance of normal blood cholesterol levels. The claim may be used only for food, which is high in unsaturated fatty acids, as referred to in the claim HIGH UNSATURATED FAT as listed in the Annex to Regulation (EC) No 1924/2006.”

Olive oil is a healthy superfood, and this is one of the primary reasons that consumers purchase it. The use and application of the NutriScore algorithm on olive oil, without consideration of its numerous health benefits, would substantially negatively impact its use and consequently primary prevention through a healthy nutrition. Currently, Mediterranean nutrition, of which olive oil is the central food, has over the decades stood the test of time as the healthiest nutritional paradigm. A lot of scientific evidence has accumulated over the last 50 years about the direct and indirect health benefits of olive oil. Such well-recognized health benefits are universally accepted by physicians, nutritionists, and dietitians (3). The evidence concerning the health benefits of olive oil have been demonstrated for cardiovascular and metabolic systems, cancer prevention, high blood pressure, cholesterol levels, cognitive/neurological conditions, diabetes, inflammatory process, oxidative stress, and coagulation, etc., (3).

Specific foods and nutritional models or diets have been even directly associated to health benefits in the frame of individual commitment toward health—the path to “personalized nutrition” described by Renna et al.—and to environmental sustainability within a “One Health” perspective (as addressed by the Med Index). This broad topic has been proposed by Kim et al., describing the traditional Korean diet composed of a multigrain rice-containing meal with fruits and nuts, and by Trichopoulou in her paper about the healthy properties of and sustainable production granted by the Greek Mediterranean Diet, with a specific focus on extra-virgin olive oil (EVO), as recently presented at the 1st International Symposium on Gastronomy and Culture, which was organized in Crete by the Yale School of Public Health. The issue of the transferability of Mediterranean nutritional habits to the non-Mediterranean is the challenge addressed by Vetrani et al. in their paper “*Planeterranea: an attempt to broaden the beneficial effects of the Mediterranean diet worldwide*,” which is aimed at developing country-specific nutritional pyramids based on the foods locally available that present the same nutritional properties and health benefits of the Mediterranean Diet (MD).

This Research Topic is very interesting and has been addressed during the “Dean's Lecture” held by Trichopoulou at the Yale School of Public Health on April 18th 2023. On the one hand, the scientific community has recognized the health benefits granted by the nutritional profile of the nutritional habits typical of Mediterranean areas and the sustainability of food production (produced close to the place where they are consumed) that preserves biodiversity and natural resources, as well as cultures and traditions. This also means that it cannot be hypothesized to export Mediterranean products and culture to other areas of the world, but the same effects in terms of the reduced prevalence of cardiovascular, metabolic, or neurodegenerative diseases as well as for cancer prevention can be obtained by proposing the consumption of local products (and healthy cooking methods) that present the same nutritional properties of foods that are typical of Mediterranean tradition. This challenge is currently being addressed by the UNESCO Chair on Health Education and Sustainable Development at the University of Naples. “Planeterranean” is the name of this newly proposed dietary model, which is also consistent with the United Nations Sustainable Development Goals (SDGs, Agenda 2030) from the perspective of the circular economy. The “Planeterranean” model could be a solution to overcome the problem of the poor nutritional model (both in term of food quality and variety) that characterizes the majority of people across the world, who receive most of their energy intake from foods with high glycemic index (such as white rice and potatoes) and sugar-rich and fatty ultra-processed foods (ready-to-eat foods, sugar-sweetened beverages, candies, chips, and pastries, etc.). These are the nutritional habits—unfortunately now frequent also in Mediterranean areas—that are responsible for the current obesity epidemic affecting both adults and children, resulting in metabolic and cardiovascular diseases or premature mortality. On the other hand, in every place of the world, it is possible to find specific fruits, vegetables, legumes, wholegrain, and sources of unsaturated fats that are consistent with the nutritional principles found to produce health benefits in the studies carried out on the Mediterranean Diet. On this basis, the UNESCO Chair has defined multiple “nutritional pyramids”—based on the foods available at the local level in different parts of the world—presenting the same nutritional properties and health benefits (and also environmentally friendly production processes) observed in the Mediterranean Diet. The research is open, as is the call for fostering healthier eating habits worldwide.

## Author contributions

All authors conceived, wrote, and approved the manuscript.
